# Development of an Autophagy-Related Gene Prognostic Model and Nomogram for Estimating Renal Clear Cell Carcinoma Survival

**DOI:** 10.1155/2021/8810849

**Published:** 2021-02-18

**Authors:** Ying Wang, Yinhui Yao, Jingyi Zhao, Chunhua Cai, Junhui Hu, Yanwu Zhao

**Affiliations:** ^1^Department of Pharmacy, The Affiliated Hospital of Chengde Medical College, Chengde 067000, China; ^2^Department of Functional Center, Chengde Medical College, Chengde 067000, China; ^3^Department of Medical Insurance, The Affiliated Hospital of Chengde Medical College, Chengde 067000, China

## Abstract

**Background:**

Kidney renal clear cell carcinoma (KIRC) is a fatal malignancy of the urinary system. Autophagy is implicated in KIRC occurrence and development. Here, we evaluated the prognostic value of autophagy-related genes (ARGs) in kidney renal clear cell carcinoma.

**Materials and Methods:**

We analyzed RNA sequencing and clinical KIRC patient data obtained from TCGA and ICGC to develop an ARG prognostic signature. Differentially expressed ARGs were further evaluated by functional assessment and bioinformatic analysis. Next, ARG score was determined in 215 KIRC patients using univariable Cox and LASSO regression analyses. An ARG nomogram was built based on multivariable Cox analysis. The prognosis nomogram model based on the ARG signatures and clinicopathological information was evaluated for discrimination, calibration, and clinical usefulness.

**Results:**

A total of 47 differentially expressed ARGs were identified. Of these, 8 candidates that significantly correlated with KIRC overall survival were subjected to LASSO analysis and an ARG score built. Functional enrichment and bioinformatic analysis were used to reveal the differentially expressed ARGs in cancer-related biological processes and pathways. Multivariate Cox analysis was used to integrate the ARG nomogram with the ARG signature and clinicopathological information. The nomogram exhibited proper calibration and discrimination (C-index = 0.75, AUC = >0.7). Decision curve analysis also showed that the nomogram was clinically useful.

**Conclusions:**

KIRC patients and doctors could benefit from ARG nomogram use in clinical practice.

## 1. Introduction

Renal cell carcinoma (RCC) accounts for 2–3% of all adult malignancies [1] and is one of the most lethal urologic cancers [2]. RCC incidence is rising in the US and most Western countries [3]. Kidney renal clear cell carcinoma (KIRC) is the most common type of RCC [4]. Despite recent treatment advances, KIRC survival is poor [5]. TMN staging is a method of determining cancer prognosis and suggesting treatment strategies. However, TMN does not consider genetic features as its classification is based on clinicopathologic information [6–8]. Thus, new markers for early KIRC detection are needed for better outcomes.

Autophagy is an evolutionarily conserved process that influences cellular homeostasis by degrading damaged organelles and intracellular content [9, 10]. Recent studies have implicated autophagy in cancer occurrence and progression [11–15]. However, whether an autophagy signature derived from one or more autophagy-related genes (ARGs) can predict long-term KIRC survival is unclear. Here, we used TCGA data to compare ARG expression profiles in KIRC vs. noncancer control tissue and assessed the ARG prognostic value. An eight-ARG prognostic signature whose prognostic value was independent of clinical factors was developed by multivariate Cox regression analyses. Next, we constructed and validated an eight-ARG prognostic model by integrating our newly established eight-ARG signature with classical clinicopathological risk factors for survival prediction in KIRC patients.

## 2. Materials and Methods

### 2.1. Autophagy-Related Genes (ARGs)

The ARGs used in this study were obtained from the Human Autophagy Database (HADb, http://www.autophagy.lu/index.html), which includes information on the 232 known autophagy genes.

### 2.2. Patient Database

ARG expression data (mRNA) and associated clinical information for KIRC patients were downloaded from TCGA. These included data on 539 KIRC tissues and 72 nontumor control tissue. Additional mRNA data on nontumor tissues from 47 patients were downloaded from the International Cancer Genome Consortium (ICGC). The following patient cases were excluded from the analysis: (a) non-KIRC cases, (b) cases lacking mRNA data, (c) cases with missing data, (d) cases with survival time <30 days, and (e) the race was white. Ultimately, 215 KIRC patients were selected for further analysis.

### 2.3. Bioinformatic Analysis

To identify differentially expressed ARGs between KIRC and nontumor samples, we used *edgeR* package on R with false discovery rate (FDR) = <0.05 and |log fold change (logFC)|>1 as cutoffs. Functional and pathway enrichment analyses were done using *clusterProfiler* package. KEGG functional pathway analysis data were visualized using the *GOplot* package. GO terms and KEGG pathways with *p = *<0.05 were considered statistically significant. Next, STRING (http://string-db.org/) and protein-protein interaction (PPI) network analyses of ARGs were done and results with a score (median confidence) > 0.4 were visualized.

Principal component analysis (PCA) was used to cluster KIRC patients into different groups using *ConsensusClusterPlus* package.

To estimate the prognostic value of ARGs, we performed univariate Cox regression analysis on the 215 KIRC patients using the *survival* package, with *p* = <0.05 indicating statistical significance. Next, least absolute shrinkage and selection operator (LASSO) Cox regression analysis was used to select potential ARGs from all significantly differentially expressed ARGs identified by univariate Cox regression analysis. LASSO Cox analysis was done using the *glmnet* package. Risk score was calculated based on a linear combination of ARG expression values after weighting regression coefficients. Patients were classified into low- and high-risk groups using median risk score as cutoff.

### 2.4. Statistical Analysis

Kaplan–Meier (KM) analysis and a two-sided log-rank test were used to determine overall survival in different clusters or in the high- and low-risk groups. Receiver operating curve (ROC) analyses using *survivalROC* package evaluated the specificity and sensitivity of prognosis prediction. Univariate and multivariate Cox regression analyses were used to evaluate correlation between prognosis and the clinicopathological features in KIRC patients.

An ARG-clinicopathologic nomogram based on multivariate analysis results was used to predict 1-, 3-, and 5-year OS. The nomogram was subjected to 1000 bootstrap resamples for internal validation of the analyzed database. Analysis of nomogram discrimination performance was determined by concordance index (C-index) analysis, which predicts the model's prognostic value. Calibration plots were also used to determine the nomogram's prognostic value. Nomogram calibration for 1-, 3-, and 5-year OS was done by comparing observed survival with the predicted probability. Additionally, a nomogram and calibration curve were developed on R using the package *rms*. Decision curve analyses (DCAs) were used to determine the nomogram's clinical utility by quantifying net benefit at various threshold probabilities in KIRC patients. DCA for 1-, 3-, and 5-year OS was done using *stdca* and *dca* packages. These analyses were done on R (version 3.5.3).

## 3. Results

### 3.1. Differentially Expressed Autophagy-Related Genes (ARGs)

**Figure 1:**
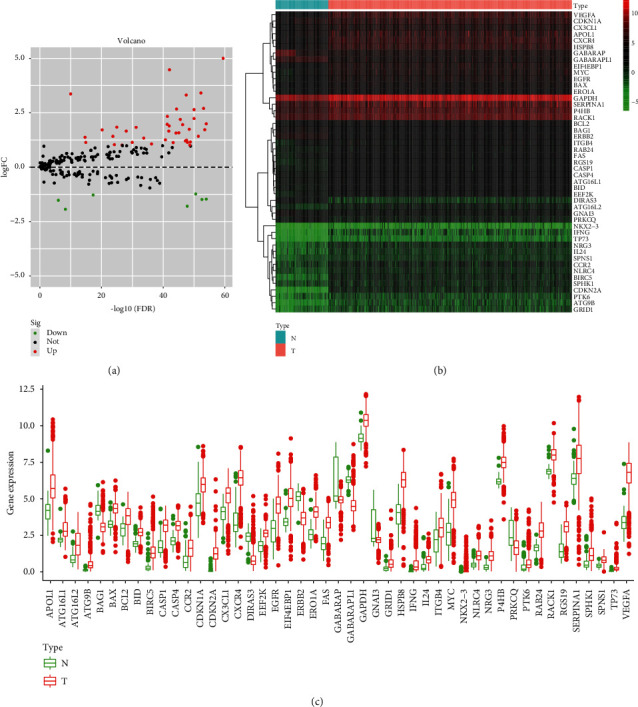
Differentially expressed autophagy-related genes (ARGs) between 119 nontumor and 539 kidney renal clear cell carcinoma (KIRC) samples. (a) The volcano plot of the 47 differentially expressed ARGs (tumor (T) vs. normal tissues (N). Red and green indicate high and low expression, respectively. (b) Hierarchical clustering of differentially expressed ARG expression levels. (c) Expression of the 47 differentially expressed ARGs.

A total of 220 ARGs were extracted and were identified to represent between 119 nontumor KIRC tissues and 539 KIRC tissues. Using FDR = <0.05 and |log (FC) | >1 as cutoffs, we identified 47 differentially expressed ARGs (tumor vs. normal tissues). Of these, 40 were upregulated and 7 were downregulated and were visualized on a scatter plot (Figures 1(a) and 1(b)).

### 3.2. Functional Annotation and Protein-Protein Interaction (PPI) Analysis

**Figure 2:**
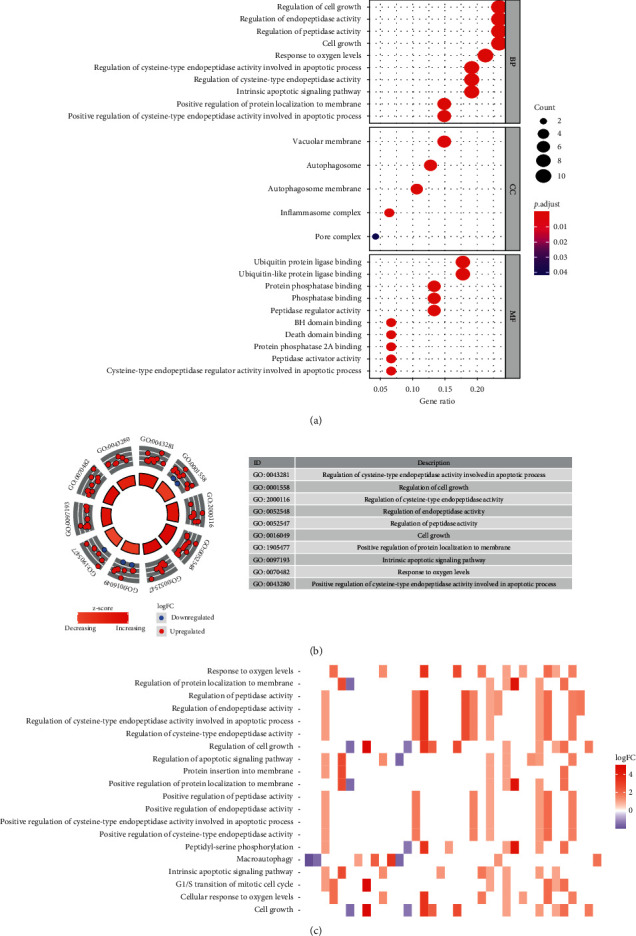
Functional annotation of the 47 differentially expressed ARGs. (a) Gene ontology analysis predicted relevant biological processes. (b) Outer circle shows a scatter plot for each term's logFC of the ARGs. (c) Heatmap of the relationship between ARGs and KEGG pathways.

The 47 differentially expressed ARGs were subjected to GO and KEGG pathway analyses to determine their biological functions. This analysis identified the top enriched terms in biological processes (BPs) as regulation of endopeptidase activity, regulation of peptidase activity, and regulation of cysteine-type endopeptidase activity involved in apoptotic process. The most enriched terms for cellular components (CCs) were autophagosome, autophagosome membrane, and inflammasome complex. The most enriched terms for molecular function (MF) were ubiquitin protein ligase binding, ubiquitin-like protein ligase binding, and peptidase regulator activity (Figure 2(a)). KEGG analysis found the 47 differentially expressed ARGs to be highly associated with human cytomegalovirus infection, autophagy-animal, and HIF-1 signaling, among other pathways. Furthermore, the z-score of enriched pathways more than zero showed that most pathways were likely to be increase (Figures 2(b) and 2(c)).

**Figure 3:**
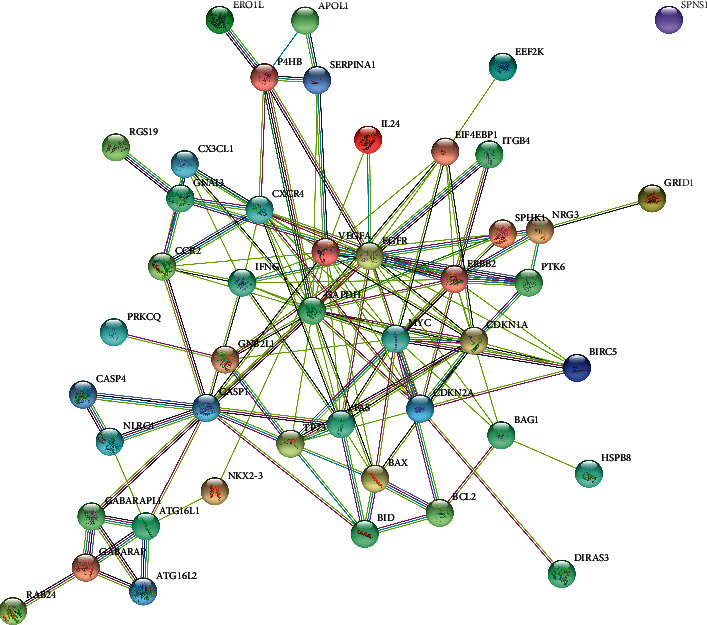
Protein-protein interaction (PPI) network of ARGs that are differentially expressed in KIRC.

Protein-protein interaction (PPI) network analysis between the 47 differentially expressed ARGs was done using STRING (Figure 3).

### 3.3. Consensus Clustering

**Figure 4:**
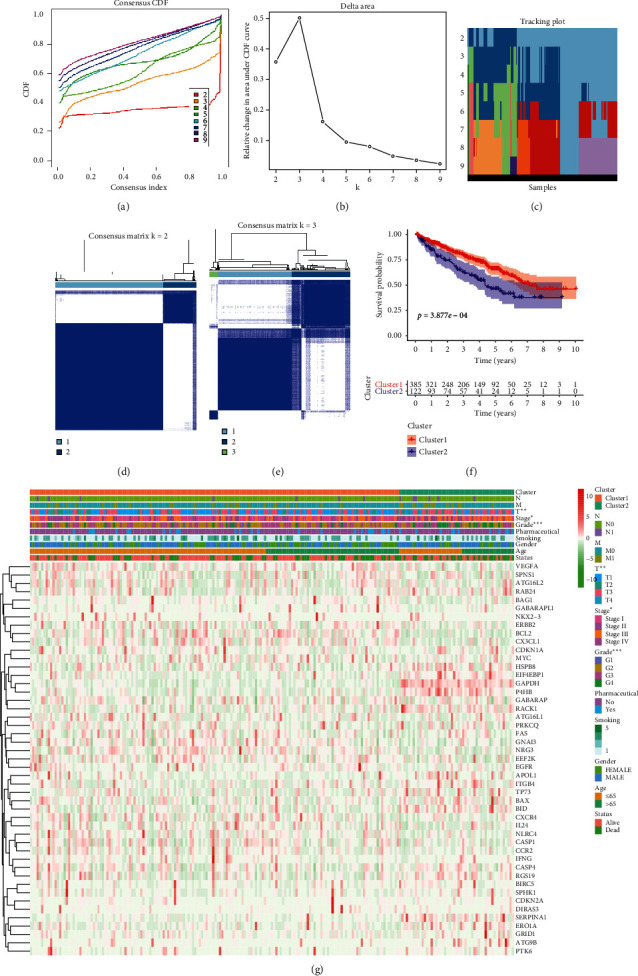
Differential clinicopathological characteristics and overall survival (OS) in KIRC clusters 1 and 2. (a) Consensus clustering cumulative distribution function (CDF) for *k* = 2 to 10. (b) Relative change in area under CDF curve for *k* = 2 to 10. (c) Tracking plot for *k* = 2 to 10. (d-e) Consensus clustering matrix for *k* = 2 (d) and *k* = 3 (e). (f) Heatmap and clinicopathological characteristics of the 2 clusters (cluster1 and cluster2) defined by ARG consensus expression. (g) Kaplan–Meier curves for KIRC patients.

To comprehend the distinct clusters of ARGs with KIRC patients, consensus clustering was performed to identify selection of adequate groups. We found that *k* = 2 was up to the mustard of clustering stability (Figures 4(a)–4(e)). Thus, KIRC patients could be grouped into 2 clusters (cluster1 and cluster2). Comparison of the 2 clusters based on KIRC patient clinicopathological features found no significant correlation between KIRC molecular clusters and clinicopathological factors such as age, gender, smoking, pharmaceutical, and pathological N or M. Notably, cluster1 significantly correlated with lower grade (*p* < 0.0001), stage (*p* < 0.05), or pathological T (*p* < 0.001) (Figure 4(g)). Kaplan–Meier survival analysis of the 2 subgroups found significant prognostic differences between KIRC patients (*p* < 0.0001), and cluster1 significantly correlated with better OS relative to cluster2 (Figure 4(f)).

### 3.4. Correlation between ARGs and KIRC

Spearman analysis of the correlation between the 47 differentially expressed ARGs and KIRC and principal component analysis (PCA) revealed a clear-cut distinction between cluster1 and cluster2 (see Supplementary 1).

### 3.5. Autophagy-Related Gene Score Building

**Figure 5:**
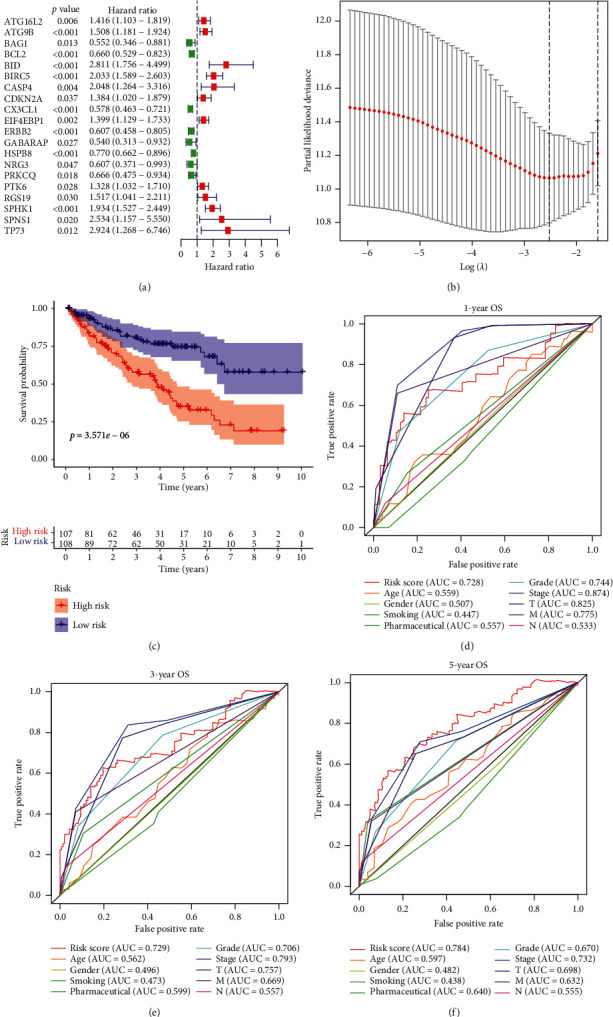
Determination of risk scores for 215 KIRC patients using the 8-ARG risk signature. (a) Identification of a 20-ARG risk signature. (b) Least absolute shrinkage and selection operator (LASSO) coefficient profiles of the 20 ARGs. (c) Kaplan–Meier survival analysis based on the 8-ARG signature risk scores in KIRC patients. (d-f) Receiver operating characteristic (ROC) curves reveal the predictive accuracy of 1-, 3-, and 5-year survival in KIRC patients.

**Figure 6:**
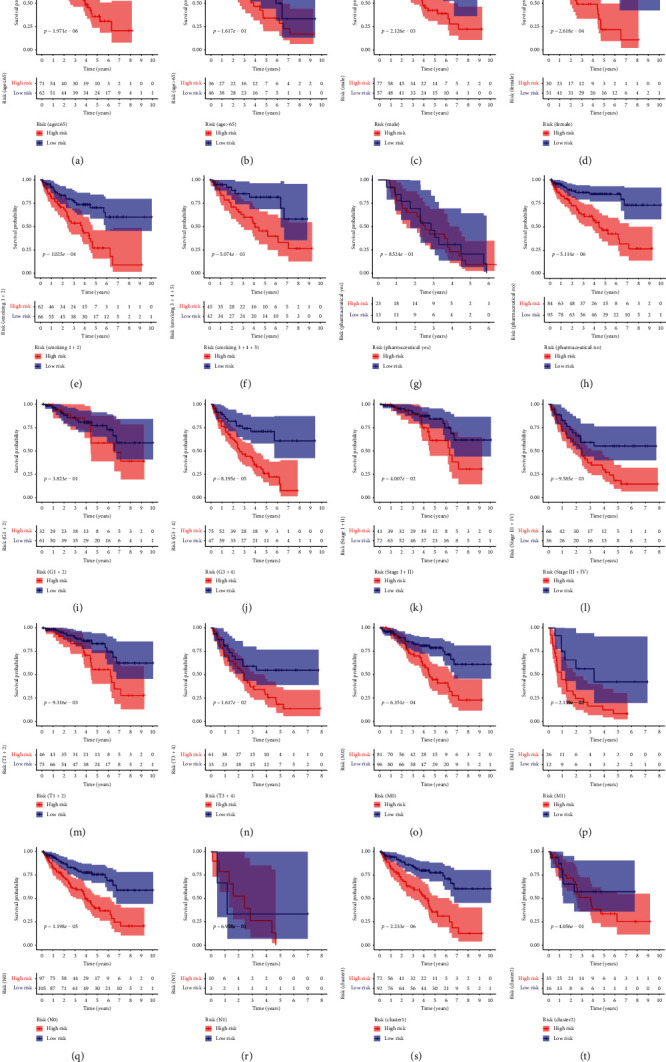
Survival differences between high- and low-risk KIRC patients stratified by clinicopathological characteristics. (a), (b) Age; (c), (d) gender; (e), (f) smoking; (g), (h) pharmaceutical; (i), (j) pathological grade; (k), (l) pathological stage; (m), (n) pathological T; (o), (p) pathological M; (q), (r) pathological N; (s), (t) cluster.

Here, we assessed the prognostic value of the 47 differentially expressed ARGs in KIRC using univariate Cox regression analysis. This analysis indicated that 20 of the forty-seven genes were strongly significantly associated with survival (*p*=<0.05). Of these 20 ARGs, 12 ARGs were associated with poor OS (hazard ratio = >1). The rest were associated with favorable OS (hazard ratio = <1) (Figure 5(a)). Finally, LASSO Cox regression analysis identified 8 ARGs (ATG16L2, ATG9B, BID, BIRC5, CX3CL1, ERBB2, HSPB8, and SPHK1) as capable of predicting KIRC clinical outcomes (Figure 5(b)). The selected 8 ARGs were then used to create a risk assessment model and risk score determined as follows: risk score = 0.028 × expression level of ATG16L2 + 0.032 × expression level of ATG9B + 0.047 × expression level of BID + 0.044 × expression level of BIRC5 + (−0.0036) × expression level of CX3CL1 + (−0.012) × expression level of ERBB2 + (−0.00099) × expression level of HSPB8 + 0.047 × expression level of SPHK1. Next, risk scores were used to group the 215 patients into high- and low-risk groups based on median risk score. KM analysis revealed significant OS differences between the 2 groups (*p* = <0.001; Figure 5(c)). To further explore utility of risk scores based on ARG signature, the 215 patients were classified into 10 subgroups based on different patient clinicopathological features. KM analysis showed that KIRC patients in the low-risk group had significantly better OS relative to those in the high-risk group in the 10 subgroups (*p* = <0.001, Figure 6).

### 3.6. Evaluation of the Predictive Performance of the Autophagy-Related Gene (ARG) Signature Using ROC Analysis

Receiver operating characteristic (ROC) curve analysis was used to evaluate the predictive accuracy of 1-, 3-, and 5-year survival in KIRC patients. The AUC values for ROC curve analysis of 1-, 3-, and 5-year ARG-based OS were 0.728, 0.729, and 0.784, respectively (Figures 5(d)–5(f)), indicating that ARG risk scores outperform conventional clinical prognostic factors in predicting long-term (5-year) but not short-term survival (1- and 3-year) in KIRC patient OS, highlighting ARG risk score as a novel KIRC prognosis indicator.

### 3.7. Establishment of the Nomogram

**Figure 7:**
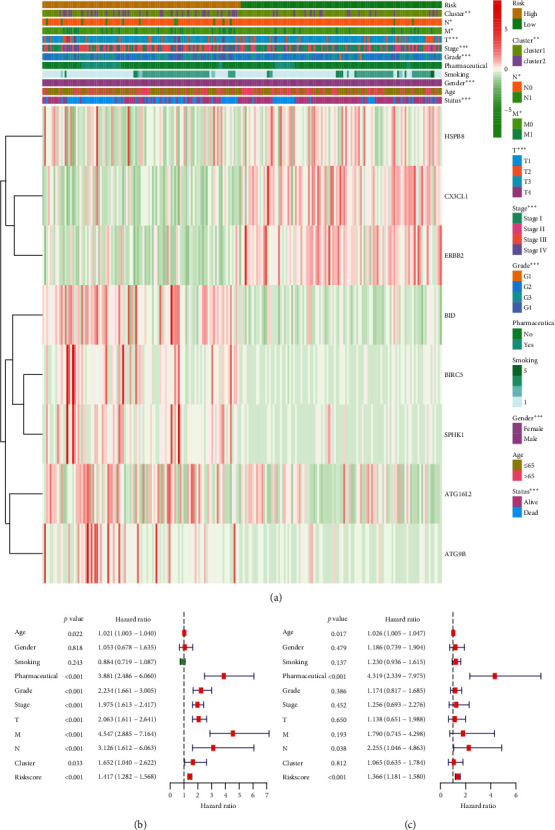
Relationship between the risk score, clinicopathological features, and cluster1/2 subgroups in 215 KIRC patients. (a) The heatmap of the 8-ARG expression in low- and high-risk KIRC. Clinicopathological feature distribution was compared in low- vs. high-risk groups. ^*∗∗*^*p* = <0.01; ^*∗∗∗*^*p* = <0.001. Forest plot of univariate (b) and multivariate (c) Cox regression analyses in KIRC.

Heatmap analysis of the expression of the 8 ARGs in high- vs. low-risk groups revealed significant differences in status (*p* *=* <0.001), gender (*p* *=* <0.001), pathological grade (*p* *=* <0.001), pathological stage (*p* *=* <0.001), pathological T (*p* = *p* < 0.001), pathological M (*p* *=* <0.05), pathological N (*p* *=* <0.05), and cluster (*p* *=* <0.01) (Figure 7(a)). Moreover, univariate Cox regression analyses of the 8-ARG signature as an independent KIRC prognostic factor showed that age, pharmaceutical, pathological grade, pathological stage, pathological T, pathological M, pathological N, cluster, and risk score correlated with significant OS differences in KIRC patients (Figure 7(b)). Multivariate analysis using the factors mentioned earlier revealed that age, pharmaceutical, pathological N, and risk score remained significantly associated with the OS (Figure 7(c)).

**Figure 8:**
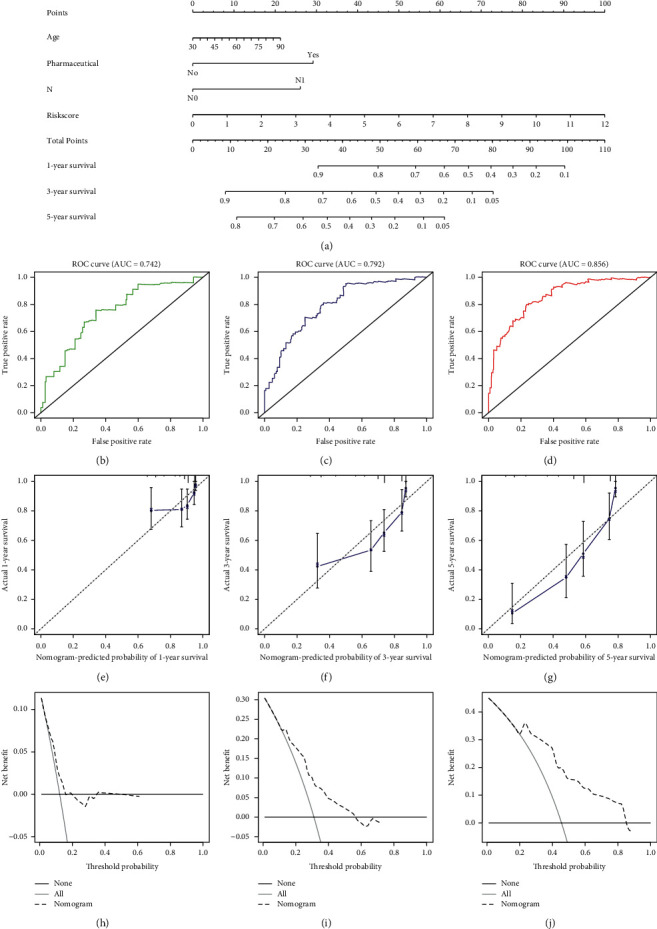
Nomogram prediction of overall survival (OS) in KIRC patients. (a) The prognostic nomogram for predicting 1-, 3-, and 5-year OS. (b–d) ROC curve based on the prognostic nomogram for 1-, 3-, and 5-year OS. (e–g) Calibration plots for predicting patient 1-, 3-, and 5-year OS. (h–j) Decision curve analyses (DCAs) of the prognostic nomogram for 1-, 3-, and 5-year risk.

A prognostic nomogram to predict 1-, 3-, and 5-year OS was established using multivariate analysis results. Total points were calculated by integrating risk score, age, and pharmaceutical (Figure 8(a)). Considering the discrimination ability of the prognostic nomogram, ROC analysis was conducted. The results indicated that AUC for 1-, 3-, and 5-year survival were 0.742, 0.792, and 0.856, suggesting that the prognostic nomogram has higher prediction efficacy (Figures 8(b)–8(d)). Moreover, the prognostic nomogram's C-index value of 0.75 (95% CI, 0.69–0.80) in all KIRC patients also indicated good discrimination.

Calibration curve analysis of the nomogram's 1-, 3-, and 5-year survival prediction revealed satisfactory predictive accuracy by the nomograms relative to actual observations (Figures 8(e)–8(g)). The nomogram's 1, 3, or 5-year decision curve analyses (DCA) showed that the more clinically useful nomogram constructs predicted long-term survival, especially 5-year survival, suggesting that if a patient or doctor's threshold probability was less than 83%, using the nomogram to predict 3- to 5-year prognosis has more benefit than completely ignoring the scheme for all programs (Figures 7(h)–7(j)). However, 1-year DCA showed a limited threshold probability range of about 18% only, indicating that the prognostic nomogram was clinically useful.

## 4. Discussion

Patient prognosis influences treatment decisions [16, 17]. ARGs have been implicated in numerous cancers, including KIRC. In past studies, some ARGs have emerged as potential KIRC prognostic factors [18–20]. For instance, BIRC5 is a crucial antiapoptotic protein that positively correlates with KIRC pathological grade and clinical stage [18]. As a molecular marker of tumor behavior and prognosis, ATG16L2 is associated with KIRC risk and patient outcome [19]. BID is located on chromosome 22q11.21 and is an apoptosis-related protein. CASP4 is reported to promote cell migration by influencing actin cytoskeleton remodeling [20]. SPHK1 upregulation in renal cell carcinoma may promote cancer progression, and its silencing may suppress cell proliferation via reduced HIF-2*α* expression [21]. ATG9B expression significantly correlates with TNM staging, distant metastasis, and survival time of clear cell renal cell carcinoma patients [22]. However, there is no consensus regarding its satisfactory predictive performance due to limited sample size or lack of data validating candidate ARGs as diagnostic and prognostic biomarkers.

Numerous studies based on TCGA datasets show that ARGs can predict OS in various cancers, including glioma [23, 24], ovarian [25], breast [26], bladder [27], and colorectal [28] cancer. Here, high-throughput RNA-seq data from TCGA were analyzed to investigate the role of ARGs in KIRC. In this study, we have constructed a useful nomogram associated with the prognostic significance of ARG scores and clinicopathologic information that can predict KIRC patient survival. In differentially expressed ARGs, many potential confounding factors were identified and established high-risk and low-risk groups, which were significantly related to OS of KIRC. Additionally, the clinicopathologic factors of age, pharmaceutical, and pathological N were integrated into the prognostic nomogram, creating a relatively accurate tool for predicting KIRC patients' OS.

Our internal validation findings indicated the nomogram's convincing discrimination and calibration power. Furthermore, an interval validation C-index = >0.7 confirmed the nomogram's clinical prognostic accuracy. However, it is still hard to determine when to use the prognostic nomogram. DCA selects the best treatment approach by analyzing various potential strategies, thereby guiding clinical decisions [29, 30]. Here, we evaluated if the prognostic nomogram could guide clinical decisions and improve patient outcomes. The 5-year decision curve analysis showed high tolerance and threshold probability (up to 83%), indicating that using the prognostic nomogram to predict long-term survival enhanced patient benefits.

Although the prognostic nomogram performs well in predicting KIRC prognosis, this study has several limitations. First, the patients in this cohort were not representative of all races affected by KIRC as the data were exclusively obtained from TCGA and ICGC databases. Secondly, because publicly available data are limited, clinicopathological characteristics were not analyzed comprehensively. Thus, while the utility of the prognostic nomogram was assessed comprehensively by an internal validation using a bootstrap test, external validation was not applied. Hence, our findings should be evaluated in prospective clinical studies.

## 5. Conclusions

In conclusion, our study not only uncovered a novel 8-gene signature as a potential biomarker of KIRC prognosis but also provided a risk assessment model for KIRC prognosis.

## Data Availability

Data underlying this study are provided in Supplementary Materials (Supplementary 2 and 3) and are available on TCGA (https://gdc.cancer.gov/) and the International Cancer Genome Consortium (ICGC) (https://icgc.org/).

## Supplementary Materials

Supplementary Materials Supplementary 1 (Figure S1): correlation between ARGs and KIRC patients. (A) Spearman correlation analysis of the 47 differentially expressed ARGs. (B) Principal component analysis of total RNA expression profiles in KIRC patients. Supplementary 2: based on the primary filter criteria mentioned in materials and methods, we identified 220 ARGs. Supplementary 3: based on the primary filter criteria mentioned in materials and methods, the clinicopathological features of 215 KIRC patients were extracted.
