# Supplementary material for: Development of an Autophagy-Related Gene Prognostic Model and Nomogram for Estimating Renal Clear Cell Carcinoma Survival
Source: J Oncol. 2021 Feb 18;2021:8810849. doi: 10.1155/2021/8810849 (PMC7910047; doi:10.1155/2021/8810849)
Supplement: Supplementary Materials — Supplementary 1 (Figure S1): correlation between ARGs and KIRC patients. (A) Spearman correlation analysis of the 47 differentially expressed ARGs. (B) Principal component analysis of total RNA expression profiles in KIRC patients. Supplementary 2: based on the primary filter criteria mentioned in materials and methods, we identified 220 ARGs. Supplementary 3: based on the primary filter criteria mentioned in materials and methods, the clinicopathological features of 215 KIRC patients were extracted. [file 8810849.f1.zip › 8810849.f1/Supplementary 1 (1).doc]

**Figure S1. Correlation between ARGs and KIRC patients** (A) Spearman correlation analysis of the 47 differentially expressed ARGs. (B) Principal component analysis of the total RNA expression profile in KIRC patients.
